# Covid-19 and gender: lower rate but same mortality of severe disease in women—an observational study

**DOI:** 10.1186/s12890-021-01455-0

**Published:** 2021-03-20

**Authors:** Federico Raimondi, Luca Novelli, Arianna Ghirardi, Filippo Maria Russo, Dario Pellegrini, Roberta Biza, Roberta Trapasso, Lisa Giuliani, Marisa Anelli, Mariangela Amoroso, Chiara Allegri, Gianluca Imeri, Claudia Sanfilippo, Sofia Comandini, England Hila, Leonardo Manesso, Lucia Gandini, Pietro Mandelli, Martina Monti, Mauro Gori, Michele Senni, Ferdinando Luca Lorini, Marco Rizzi, Tiziano Barbui, Laura Paris, Alessandro Rambaldi, Roberto Cosentini, Giulio Guagliumi, Simonetta Cesa, Michele Colledan, Maria Sessa, Arianna Masciulli, Antonello Gavazzi, Sabrina Buoro, Giuseppe Remuzzi, Piero Ruggenenti, Annapaola Callegaro, Andrea Gianatti, Claudio Farina, Antonio Bellasi, Sandro Sironi, Stefano Fagiuoli, Fabiano Di Marco, HPG23 Covid-19 Study Group

**Affiliations:** 1 Pulmonary Medicine Unit, Medicine Department, ASST Papa Giovanni XXIII, Piazza OMS, 1, 24127 Bergamo, Italy; 2 Università degli Studi di Milano, Milan, Italy; 3 FROM Research Foundation, Bergamo, Italy; 4 Intensive Care Unit, ASST Papa Giovanni XXIII, Bergamo, Italy; 5 Cardiovascular Department, ASST Papa Giovanni XXIII, Bergamo, Italy; 6 Infectious Diseases Unit, ASST Papa Giovanni XXIII, Bergamo, Italy; 7 Department of Oncology and Hematology, ASST Papa Giovanni XXIII, Bergamo, Italy; 8 Emergency Department, ASST Papa Giovanni XXIII, Bergamo, Italy; 9 Department of Organ Failure and Transplantation, ASST Papa Giovanni XXIII, Bergamo, Italy; 10 Department of Health and Social Care Professions, ASST Papa Giovanni XXIII, Bergamo, Italy; 11 Neurology Unit, ASST Papa Giovanni XXIII, Bergamo, Italy; 12 Quality Management, ASST Papa Giovanni XXIII, Bergamo, Italy; 13 Mario Negri Institute for Pharmacological Research IRCCS, Anna Maria Astori Centre, Science and Technology Park Kilometro Rosso, Bergamo, Italy; 14 Nephrology Unit, ASST Papa Giovanni XXIII, Bergamo, Italy; 15 Microbiology and Virology Unit, ASST Papa Giovanni XXIII, Bergamo, Italy; 16 Pathology Unit, ASST Papa Giovanni XXIII, Bergamo, Italy; 17 Department of Research, Innovation, Brand Reputation, ASST Papa Giovanni XXIII, Bergamo, Italy; 18 Department of Diagnostic Radiology, ASST Papa Giovanni XXIII, Bergamo, Italy; 19 Università degli Studi di Milano-Bicocca, Milan, Italy; 20 Gastroenterology Hepatology and Transplantation Unit, ASST Papa Giovanni XXIII, Bergamo, Italy

**Keywords:** Covid-19, Gender, Disease severity

## Abstract

**Background:**

Gender-related factors might affect vulnerability to Covid-19. The aim of this study was to describe the role of gender on clinical features and 28-day mortality in Covid-19 patients.

**Methods:**

Observational study of Covid-19 patients hospitalized in Bergamo, Italy, during the first three weeks of the outbreak. Medical records, clinical, radiological and laboratory findings upon admission and treatment have been collected. Primary outcome was 28-day mortality since hospitalization.

**Results:**

431 consecutive adult patients were admitted. Female patients were 119 (27.6%) with a mean age of 67.0 ± 14.5 years (vs 67.8 ± 12.5 for males, *p* = 0.54). Previous history of myocardial infarction, vasculopathy and former smoking habits were more common for males. At the time of admission PaO_2_/FiO_2_ was similar between men and women (228 [IQR, 134–273] vs 238 mmHg [150–281], *p* = 0.28). Continuous Positive Airway Pressure (CPAP) assistance was needed in the first 24 h more frequently in male patients (25.7% *vs* 13.0%; *p* = 0.006). Overall 28-day mortality was 26.1% in women and 38.1% in men (*p *= 0.018). Gender did not result an independent predictor of death once the parameters related to disease severity at presentation were included in the multivariable analysis (*p* = 0.898). Accordingly, the Kaplan–Meier survival analysis in female and male patients requiring CPAP or non-invasive ventilation in the first 24 h did not find a significant difference (*p* = 0.687).

**Conclusion:**

Hospitalized women are less likely to die from Covid-19; however, once severe disease occurs, the risk of dying is similar to men. Further studies are needed to better investigate the role of gender in clinical course and outcome of Covid-19.

**Supplementary Information:**

The online version contains supplementary material available at 10.1186/s12890-021-01455-0.

## Background

Severe Acute Respiratory Syndrome Coronavirus (SARS-CoV-2) infection disease (Covid-19) was first described in December 2019 in Whuan, China. As of 12 March 2020, due to the growing number of countries involved, the World Health Organization (WHO) declared Covid-19 a pandemic [1]. Northern Italy has been one of the first and most severely affected area of Europe, with an increase in mortality up to + 568% in Bergamo and its Province (over 1 million inhabitants), in March 2020 compared to the same month in 2015–2019 [2].

A growing body of literature on SARS-CoV-2 infection addressing immediate biomedical needs (e.g. clinical characteristics, mortality, and predictors of outcome) is becoming available, however only few articles specifically refer to the gender dimension of Covid-19 [3–7]. In the 2003 Severe Acute Respiratory Syndrome (SARS, caused by SARS-CoV-1 infection) epidemic, sex differences indicated a lower risk of death in women [8]. Analogous observations can be made for SARS-CoV-2 infection for which, compared to women, men have a higher mortality risk [7, 9–12]. These results suggest an underlying sex-dependent susceptibility. An enzymatic system involved in this different sex predisposition could be represented by angiotensin converting enzyme 2 (ACE2), which is the functional receptor for SARS-CoV-1 and 2 [13]. In animal experimental models ACE2 expression is found to be influenced by sex hormones; in female mice, ovariectomy or administration of estrogen-receptor antagonists increased the mortality of SARS-CoV-1 infection [14]. Furthermore, women have stronger innate and adaptive immunity and overall greater resistance to viral infections than men [15]. As emphasized for previous outbreaks of global concern (i.e. MERS, H1N1, H5N1, SARS, Zika and Ebola virus), the study of gender dimension, which is both physical and social constructed, is important to understand the pathogenic mechanisms and to eventually design better therapeutic strategies [16].

The aim of this study was to describe the role of gender in terms of clinical features and 28-day outcomes of hospitalized Covid-19 patients.

## Methods

This retrospective, observational study was approved by the local Ethics Committee (Comitato Etico di Bergamo, Italy. N°37/2020). In the light of the urgent need to treat critical patients, and to avoid paper contamination, verbal consent was obtained when feasible, according to local protocol.

### Source of data

We collected data from electronic medical records of all adult patients with laboratory-confirmed SARS-CoV-2 infection, consecutively hospitalized for clinical reasons (i.e. respiratory failure in almost all cases) at Papa Giovanni XXIII Hospital (a tertiary hospital of 1080 beds), and its affiliate hospital, San Giovanni Bianco (a community hospital of 130 beds), between February 23rd and March 14th, 2020. Follow-up stopped on April 11th, 2020, to allow the observation for a minimum of 28 days in all patients since hospitalization. We did not include patients under eighteen year-old or patients already hospitalized for other conditions. Covid-19 has been diagnosed on the basis of the updated WHO interim guidance [17]. Medical history, demographic data, underlying comorbidities, viral exposure, clinical symptoms and/or signs, radiological and laboratory findings upon admission were derived from medical records, whereas information about family unit, healthcare job, pre-hospital medical contact, use of antibiotics and flu vaccine status were self-reported by the patient or relatives. Immunosuppression was defined as iatrogenic when due to chemotherapy, or treatment for solid organ transplantation or autoimmune diseases, otherwise it was HIV-related (Human Immunodeficiency Virus). Radiologic assessments and all laboratory tests were performed according to local clinical practice and based on clinical needs. At presentation, patients underwent routine blood tests, arterial blood gas analysis (ABG), and chest X-ray.

### Laboratory confirmation of SARS-CoV-2 infection

SARS-CoV-2 genome from nasal swabs and respiratory samples was detected by two different molecular methods (GeneFinder COVID-19-Elitech Group, Allplex™ 2019—nCoV Assay—Seegene Inc) in line with the manufacturer’s instructions. After the purification of viral RNA from clinical samples, the detection of RdRp, E and N viral genes was obtained by real time Polymerase Chain Reaction (RT-PCR) according to WHO protocol [18].

### Outcomes

The aim of this study was to describe gender differences in terms of clinical features and 28-day outcomes since hospitalization. The primary endpoint was 28-day all-cause mortality, occurring either during in-hospital stay or after discharge. The secondary endpoint was the development of severe disease, a composite outcome defined as the occurrence of at least one of the following: intensive or respiratory sub-intensive care unit admission; need of endotracheal intubation (ETI) and invasive ventilation, non-invasive ventilation (NIV), or continuous positive airway pressure (CPAP); death during hospitalization or after discharge.

### Statistical analysis

Descriptive statistics were used to summarize the baseline characteristics of Covid-19 patients. Continuous variables were expressed as mean ± standard deviation (SD) or as median and interquartile range [IQR], depending on their parametric or non-parametric distribution. Categorical variables were expressed as absolute counts and percentages. The chi-square test (or Fisher’s exact test when appropriate) was used to test between group differences for the categorical variables, whereas the t-test or the Wilcoxon–Mann–Whitney test (for normally and not normally distributed variables, respectively) were used to compare continuous variables. Survival curve (overall and stratified by the need of CPAP/NIV at entry), according to gender was reported, with comparison by the log-rank test. Univariate logistic regression model was run to investigate predictors of 28-day mortality. A backward stepwise procedure was used to determine the best predictors of mortality to be included in the multivariable model. Results are presented as odds ratio (OR) with 95% confidence intervals (CI). Candidate predictors included in the stepwise procedure were variables that were available in at least 65% of patients and significantly different between patients who died and those who did not at a *p* value level of 0.05. The final multivariable model included predictors selected from the stepwise procedure along with some few other variables selected on biological plausibility and clinical judgment. To overcome the constraint of biased/overestimated results that may arise as a result of missing data, multiple imputation by chained equation (MICE), with 20 imputation sets, was used to impute the missing covariates involved in the final multivariable model. For all tested hypotheses, a two‐tailed *p* values < 0.05 was considered significant. Analyses were performed using STATA software, release 16 (StataCorp LP, College Station, TX, USA).

## Results

In the first three weeks of the pandemic 431 adult Covid-19 patients were admitted by the ER to our hospital. Their demographic and clinical characteristics are reported in Table 1. 119 were female (27.6%), and the majority were Caucasian (98.6%). The mean (± SD) age was 67.6 ± 13.0 years, notably no significant differences were observed between female and male (67.0 ± 14.5 vs 67.8 ± 12.5 years, respectively, *p* = 0.54). Regardless of gender, most patients had documented relevant comorbidities, especially systemic hypertension (55.8%), and diabetes (19.8%). However, being a former smoker, a previous history of myocardial infarction or vasculopathy were significantly more common in males (33.3% *vs* 20.5%, 16.1% *vs* 3.4%, 15.4% *vs* 6.8%; *p* = 0.025, *p* < 0.001, *p* = 0.020; respectively). The comorbidity burden, as estimated by the Charlson Comorbidity Index (CCI), attained a median value of 4.0 [IQR 2.0–5.0] and was comparable between categories as well as medication history, immunosuppression and flu vaccine status.

**Table 1:** Demographic and clinical characteristics in all patients and by gender

	N	All patients (N = 431)	Gender		
			Female (N = 119)	Male (N = 312)	*p*
Age—y mean (SD)	431	67.6 (13.0)	67.0 (14.5)	67.8 (12.5)	0.54
≤ 59—no. (%)	431	109 (25.3)	32 (26.9)	77 (24.7)	0.29
60–69—no. (%)		103 (23.9)	24 (20.2)	79 (25.3)	
70–77—no. (%)		114 (26.5)	38 (31.9)	76 (24.4)	
≥ 78—no. (%)		105 (24.4)	25 (21.0)	80 (25.6)	
Caucasian ethnicity—no. (%)	431	425 (98.6)	118 (99.2)	307 (98.4)	0.55
BMI^a^—median [IQR]	359	26.8 [24.5–30.2]	26.3 [22.8–31.2]	27.0 [24.8–30.1]	0.14
≥ 30—no. (%)	359	97 (27.0)	30 (30.9)	67 (25.6)	0.31
Smoking history—no. (%)					
Current smoker	319	18 (5.6)	4 (4.5)	14 (6.1)	0.60
Former smoker	319	95 (29.8)	18 (20.5)	77 (33.3)	0.025
Never smoker	319	206 (64.6)	66 (75.0)	140 (60.6)	0.016
Comorbidities—no. (%)					
Hypertension	425	237 (55.8)	61 (51.7)	176 (57.3)	0.29
Diabetes	425	84 (19.8)	18 (15.3)	66 (21.5)	0.15
Chronic Kidney Failure	423	31 (7.3)	9 (7.8)	22 (7.2)	0.83
COPD	423	41 (9.7)	13 (11.1)	28 (9.2)	0.54
Active solid neoplasm	423	16 (3.8)	6 (5.1)	10 (3.3)	0.37
Active hematologic malignancy	423	11 (2.6)	2 (1.7)	9 (2.9)	0.48
Cerebrovascular disease	422	25 (5.9)	6 (5.1)	19 (6.2)	0.67
Previous Myocardial Infarction	422	53 (12.6)	4 (3.4)	49 (16.1)	< 0.001
Chronic heart failure	423	16 (3.8)	5 (4.3)	11 (3.6)	0.74
Vasculopathy	423	55 (13.0)	8 (6.8)	47 (15.4)	0.020
Rheumatic pathology	423	28 (6.6)	9 (7.7)	19 (6.2)	0.58
CCI score—median [IQR]	426	4.0 [2.0–5.0]	4.0 [2.0–5.0]	4.0 [2.0–5.0]	0.27
CCI = 0—no. (%)	426	36 (8.5)	13 (10.9)	23 (7.5)	0.41
CCI = 1–2—no. (%)		102 (23.9)	25 (21.0)	77 (25.1)	
CCI = 3 + – no. (%)		288 (67.6)	81 (68.1)	207 (67.4)	
Medication history—no. (%)					
Antihypertensives	412	224 (54.4)	60 (52.2)	164 (55.2)	0.58
ACE-inhibitors	416	73 (17.5)	21 (18.4)	52 (17.2)	0.77
ARBs	416	72 (17.3)	13 (11.4)	59 (19.5)	0.050
Steroids	415	20 (4.8)	8 (6.9)	12 (4.0)	0.22
Oral antidiabetics	415	59 (14.2)	16 (13.8)	43 (14.4)	0.88
Insulin	415	27 (6.5)	5 (4.3)	22 (7.4)	0.26
OAT/DOACs	413	51 (12.3)	18 (15.7)	33 (11.1)	0.20
Antiplatelets	415	112 (27.0)	28 (24.1)	84 (28.1)	0.42
Long-term oxygen therapy	423	10 (2.4)	4 (3.4)	6 (2.0)	0.38
Immunosuppression—no. (%)	423	27 (6.4)	9 (7.7)	18 (5.9)	0.50
Iatrogenic	423	27 (6.4)	9 (7.7)	18 (5.9)	0.51
HIV	423	0 (0.0)	0 (0.0)	0 (0.0)	–
Flu vaccine—no. (%)	290	142 (49.0)	35 (42.7)	107 (51.4)	0.18

Data expressed as column percentages. Percentages may not total 100 because of rounding. BMI = Body Mass Index

^a^Body Weight and Height as referred by patient; COPD = Chronic Obstructive Pulmonary Disease; CCI = Charlson Comorbidity Index score; Antihypertensives: (ACE-inhibitors, ARBs [Angiotensin Receptor Blockers], Calcium channels blockers, Diuretics, Beta-blockers, Alpha-blockers, Alpha-2 agonists); OAT = Oral Anticoagulant Therapy; DOACs = Direct Oral Anticoagulants; Iatrogenic immunosuppression (due to chemotherapy, solid organ transplantation, autoimmune diseases), HIV (Human Immunodeficiency Virus); SD = Standard Deviation; IQR = Interquartile Range; *p* values obtained by Chi-square test (or Fisher's exact test when appropriate) for categorical variables and t-test (or Wilcoxon–Mann–Whitney test when appropriated) for continuous variables

### Pre-hospital, clinical and laboratory features at presentation

Pre-hospital epidemiology, in terms of contact with healthcare facilities or infection occurrence within family unit, was homogeneous among groups (Table 2). Half of the patients had taken antibiotics before hospitalization and fever was the most frequent symptom at home (90.1%), followed by dyspnea (59.3%) and cough (50.2%). Interestingly, gastrointestinal symptoms (i.e. anorexia, nausea, vomiting, diarrhea) were significantly more prevalent in females (24.6% vs 15.7%; *p* = 0.033). Relevant intervals between symptoms onset and clinically important episodes are reported in Additional file 1: Table S1. The median interval between symptoms onset and ER admission resulted of 7.0 days [5.0–10.0], without significant difference according to gender (8.0 [5.0–10.5] vs 7.0 [5.0–10.0]) in females and males, respectively, *p* = 0.97). Of note, interval between hospitalization and CPAP/NIV treatment was significantly shorter in males (1 day [1–3] vs 2 days [1–4]; *p* = 0.017).

**Table 2:** Pre-hospital epidemiology and clinical features in all patients and by gender

	N	All patients (N = 431)	Gender		
			Female (N = 119)	Male (N = 312)	*p*
Pre-hospital antibiotic^a^—no. (%)	384	192 (50.0)	49 (45.4)	143 (51.8)	0.26
Contact with healthcare facilities in the last 14 days^b^—no. (%)	380	125 (32.9)	40 (36.4)	85 (31.5)	0.36
Contact with minors in the last 14 days—no. (%)	271	99 (36.5)	33 (41.8)	66 (34.4)	0.25
Family members^c^, median [IQR]	295	2.0 [2.0–3.0]	2.0 [2.0–3.0]	2.0 [2.0–3.0]	0.85
Family member with confirmed Covid-19—no. (%)	296	45 (15.2)	15 (17.6)	30 (14.2)	0.46
Contact with Covid-19 confirmed case—no. (%)	317	81 (25.6)	21 (22.6)	60 (26.8)	0.43
Healthcare professional—no. (%)	380	27 (7.1)	8 (7.5)	19 (7.0)	0.86
Symptoms^d^—no. (%)					
Fever	425	383 (90.1)	107 (90.7)	276 (89.9)	0.81
Cough	424	213 (50.2)	62 (52.5)	151 (49.3)	0.56
Dyspnoea	425	252 (59.3)	68 (57.6)	184 (59.9)	0.66
Sore throat	424	13 (3.1)	6 (5.1)	7 (2.3)	0.20
Dizziness	424	18 (4.2)	9 (7.6)	9 (2.9)	0.032
Abdominal pain	424	10 (2.4)	5 (4.2)	5 (1.6)	0.11
Chest pain	424	15 (3.5)	3 (2.5)	12 (3.9)	0.49
Systemic (asthenia, myalgia)	424	137 (32.3)	45 (38.1)	92 (30.1)	0.11
Gastrointestinal^e^	424	77 (18.2)	29 (24.6)	48 (15.7)	0.033

Data expressed as column percentages. Percentages may not total 100 because of rounding

^a^Pre-hospital antibiotic therapy prescribed for onset of symptoms related to Covid-19

^b^Access to healthcare facilities (i.e. outpatient clinic consultations, dialysis, previous hospitalization, assistance or visits to hospitalized or in retirement home people)

^c^Family member number intended as cohabitants

^d^Symptoms referred by patients to the Emergency Room (ER) as described in admission dossier

^e^Gastrointestinal symptoms include anorexia, nausea, vomiting and diarrhea. IQR = Interquartile Range; *p* values obtained by Chi-square test (or Fisher's exact test when appropriate) for categorical variables

Characteristics at presentation to ER are shown in Table 3. Most of the patients were alert (93.8%), and febrile (65.2%), with normal blood pressure and normal heart rate. ABG showed for both sexes a tendency to respiratory alkalosis, with a median pH of 7.47 [7.44–7.50], median PaCO_2_ of 33 mmHg [IQR 30–35], and median HCO_3_^−^ of 24.1 mmol/l [22.0–26.0]. Females showed a small but significant higher heart rate and HCO_3_^−^ (Table 3). Also PaO_2_/FiO_2_ at admission, on average severely reduced, was not statistically different between male and female (229 mmHg [134–273] vs 238 mmHg [150–281], *p* = 0.28).

**Table 3:** Clinical characteristics, blood gas analysis at presentation and in-hospital treatments in all patients and by gender

	N	All patients (N = 431)	Gender		
			Female (N = 119)	Male (N = 312)	*p*
At entry in emergency room					
AVPU—no. (%)	421				
A (alert)		395 (93.8)	109 (93.2)	286 (94.1)	0.39
V (verbal)		4 (1.0)	2 (1.7)	2 (0.7)	
P (pain)		4 (1.0)	2 (1.7)	2 (0.7)	
U (unresponsive)		18 (4.3)	4 (3.4)	14 (4.6)	
HR, bpm—median [IQR]	400	84 [75–94]	87 [78–95]	83 [73–93]	0.024
SBP, mmHg—median [IQR]	395	126 [112–140]	126 [113–140]	126 [110–140]	0.82
RR, acts/min—median [IQR]	221	20 [16–26]	20 [16–26]	20 [16–26]	0.35
Fever—no. (%)	405	264 (65.2)	69 (61.1)	195 (66.8)	0.28
pH—median [IQR]	277	7.47 [7.44–7.50]	7.49 [7.44–7.51]	7.47 [7.44–7.50]	0.16
PaO_2_/FiO_2_—median [IQR]	295	229 [142–278]	238 [150–281]	229 [134–273]	0.28
< 200—no. (%)	295	113 (38.3)	23 (32.9)	90 (40.0)	0.28
PaCO_2_, mmHg—median [IQR]	292	33.0 [29.7–35.0]	32.0 [30.0–36.2]	33.0 [29.0–35.0]	0.62
HCO_3_^−^, mmol/L—median [IQR]	151	24.1 [22.0–26.0]	25.4 [23.4–27.4]	24.0 [22.0–25.0]	< 0.001
Lac, mmol/L—median [IQR]	188	1.38 [1.01–1.74]	1.26 [1.00–1.62]	1.40 [1.03–1.88]	0.27
*In the first 24 h*					
Oxygen and ventilatory support^a^—no. (%)					
Low flow oxygen nasal cannula	415	111 (26.7)	40 (34.8)	71 (23.7)	0.022
Venturi mask	415	50 (12.0)	11 (9.6)	39 (13.0)	0.34
Non-rebreather mask	415	95 (22.9)	27 (23.5)	68 (22.7)	0.86
CPAP	415	92 (22.2)	15 (13.0)	77 (25.7)	0.006
NIV	415	14 (3.4)	2 (1.7)	12 (4.0)	0.25
ETI	415	12 (2.9)	4 (3.5)	8 (2.7)	0.74
FiO_2_—median [IQR]	373	60.0 [35.0–70.0]	50.0 [30.0–70.0]	60.0 [35.0–70.0]	0.025
PEEP, cmH_2_O—median [IQR]	109	15.0 [12.0–16.0]	15.0 [13.0–15.5]	15.0 [12.0–16.0]	0.67
IPAP, cmH_2_O—median [IQR]	15	20.0 [16.0–24.0]	21.0 [20.0–22.0]	20.0 [16.0–24.0]	0.86
Antiviral^b^—no. (%)	399	326 (81.7)	83 (76.9)	243 (83.5)	0.13
Hydroxychloroquine—no. (%)	392	305 (77.8)	81 (76.4)	224 (78.3)	0.69
Steroid^c^—no. (%)	395	39 (9.9)	6 (5.6)	33 (11.5)	0.083
Antibiotics^d^—no. (%)	406	372 (91.6)	103 (92.0)	269 (91.5)	0.88
IL-6 inhibitors^e^—no. (%)	387	17 (4.4)	0 (0.0)	17 (6.0)	0.005

Data expressed as column percentages. Percentages may not total 100 because of rounding. AVPU = level of consciousness; HR = Heart Rate; RR = Respiratory Rate; SBP = Systolic Blood Pressure; ER = Emergency Room; FiO_2_ = Fraction of inspired oxygen; PaO_2_ = Partial pressure of oxygen in arterial blood; SaO_2_ = Oxygen arterial Saturation; PaCO_2_ = Partial pressure of Carbon Dioxide in arterial blood; HCO_3_^−^ = Bicarbonate; Lac = Lactate concentration in arterial blood; PEEP = Positive End Expiratory Pressure; IPAP = Inspiratory Positive Airway Pressure (Pressure support + PEEP)

^a^Oxygen and Ventilatory support in the first 24 h by the ER presentation intended as the highest between Low flow oxygen nasal cannula, Venturi mask, Non-rebreather mask (reservoir), Continuous Positive Airway Pressure (CPAP) with helmet, Non-invasive ventilation (NIV) and Endotracheal Intubation (ETI)

^b^Antiviral therapy intended as at least one of the following: Oseltamivir, Lopinavir/Ritonavir, Remdesivir and Darunavir/Cobicistat

^c^Steroid therapy intended as Methylprednisolone, Hydrocortisone and Dexamethasone

^d^Antibiotic Therapy was prescribed at Physician’s discretion and at least one of the following: Ceftriaxone, Cefixime, Azithromycin or Levofloxacin

^e^IL-6 inhibitors, intended as Tocilizumab or Siltuximab, were prescribed in according to shared local clinical protocol. IQR = Interquartile Range; *p* values obtained by Chi-square test (or Fisher's exact test when appropriate) for categorical variables and Wilcoxon-Mann–Whitney test for continuous variables

Laboratory and radiographic findings at admission are reported in Table 4. Liver transaminases, both AST and ALT, were higher in males than in females (53 U/L vs 40 U/L and 40 U/L vs 30 U/L, respectively; *p* < 0.001). Urea and creatinine values were normal, although significantly lower in female (0.74 mg/dL *vs* 0.98 mg/dL and 38 mg/dL *vs* 48 mg/dL, respectively; *p* < 0.001). CRP was generally elevated (113 [56–162] mg/L), with cases of very high levels (i.e. > 127 mg/L) being more common in males (35.4 vs 48.7%, *p* 0.015). Procalcitonin (PCT) levels were higher in males (0.56 ng/mL [IQR 0.21–2.35] vs 0.12 ng/mL [IQR 0.05–1.00]; *p* = 0.019). The complete blood count, coagulation parameters, liver and renal function, although differed from gender categories, in the majority of cases were in the range of normality. The overall prevalence of chest X-ray abnormalities at presentation were comparable in men and women, as well as the unilateral or bilateral onset of viral pneumonia (Table 4).

**Table 4:** Laboratory and radiographic findings at hospital admission in all patients and by gender

	N	All patients (N = 431)	Gender		
			Female (N = 119)	Male (N = 312)	*p*
Hemoglobin, g/L	417	134 [123–148]	127 [117–136]	139 [126–150]	< 0.001
White blood cells, 10^9^/L	417	6.25 [4.98–8.83]	5.92 [4.77–7.82]	6.44 [5.06–8.95]	0.10
Neutrophils, 10^9^/L	376	4.74 [3.55–7.21]	4.52 [3.55–6.50]	4.87 [3.55–7.32]	0.50
Neutrophils, %	376	77.7 [70.5–84.7]	76.6 [69.7–84.2]	78.0 [71.3–84.8]	0.54
Lymphocytes, 10^9^/L	263	0.89 [0.58–1.23]	0.89 [0.62–1.13]	0.90 [0.58–1.24]	0.68
Lymphocytes, %	263	14.0 [7.9–19.5]	14.6 [8.7–19.9]	13.9 [7.6–19.3]	0.56
Monocytes, %	263	6.1 [3.9–8.1]	5.2 [3.2–7.9]	6.2 [4.2–8.3]	0.048
Eosinophils, %	263	0.00 [0.00–0.30]	0.00 [0.00–0.30]	0.00 [0.00–0.30]	0.65
Basophils, %	263	0.20 [0.10–0.30]	0.20 [0.10–0.30]	0.20 [0.10–0.30]	0.75
Platelets, 10^9^/L	407	179 [140–226]	209 [158–252]	171 [136–212]	< 0.001
≥ 150—no. (%)	407	290 (71.3)	94 (83.9)	196 (66.4)	< 0.001
INR, ratio	353	1.07 [1.02–1.16]	1.05 [1.01–1.12]	1.08 [1.02–1.16]	0.037
aPTT, ratio	338	1.15 [1.04–1.29]	1.09 [0.99–1.27]	1.17 [1.06–1.30]	0.024
AST, U/L	410	49.0 [35.0–74.0]	40.0 [29.0–55.0]	53.0 [38.0–84.0]	< 0.001
ALT, U/L	412	37.0 [25.0–59.0]	30.0 [20.0–45.0]	40.0 [27.0–62.0]	< 0.001
Total bilirubin, mg/dL	372	0.60 [0.50–0.90]	0.60 [0.40–0.80]	0.70 [0.50–0.90]	0.020
Creatinine, mg/dL	414	0.92 [0.77–1.23]	0.74 [0.61–0.91]	0.98 [0.84–1.35]	< 0.001
Urea, mg/dL	354	45.0 [34.0–66.0]	38.0 [28.0–55.0]	48.0 [37.0–71.0]	< 0.001
LDH, U/L	371	384 [300–516]	368 [291–484]	391 [308–529]	0.13
D-dimer, ng/mL	13	2337 [1587–6028]	4182 [1864–11437]	2067 [1463–3328]	0.32
Fibrinogen, g/L	94	0.61 [0.49–0.71]	0.53 [0.48–0.66]	0.63 [0.49–0.72]	0.19
CRP, mg/L	417	113 [56–16.2]	102 [48–145]	122 [58–165]	0.051
≥ 127—no. (%)	417	188 (45.1)	40 (35.4)	148 (48.7)	0.015
PCT, ng/mL	84	0.49 [0.12–1.90]	0.12 [0.05–1.00]	0.56 [0.21–2.35]	0.019
Na, mEq/L	414	138 [136–141]	138 [136–140]	138 [136–141]	0.86
K, mEq/L	412	3.9 [3.6–4.3]	3.8 [3.5–4.2]	4.0 [3.7–4.3]	0.028
Cl, mEq/L	289	102 [99–104]	101 [98–103]	102 [99–104]	0.46
Abnormalities at chest X-ray—no. (%)	413	355 (86.0)	98 (87.5)	257 (85.4)	0.58
Unilateral	413	77 (18.6)	17 (15.2)	60 (19.9)	0.27
Bilateral	413	278 (67.3)	81 (72.3)	197 (65.4)	0.19

Data expressed as median and IQR [Interquartile Range] (continuous variables) and as column percentages (categorical variables). Percentages may not total 100 because of rounding, Hemocromocytometric values data shown as Hemoglobin, White blood cells and Platelets count. INR = International Normalization Ratio for prothrombin time; aPTT = Activated Partial Thromboplastin Time; AST = Aspartate Aminotransferase; ALT = Alanine Aminotransferase; LDH = Lactate Dehydrogenase; D-dimer = Fibrin Degradation product; CRP = C-reactive Protein; PCT = Procalcitonin; Na = Sodium; K = Potassium; Cl = Chloride. *p* values obtained by Chi-square test (or Fisher's exact test when appropriate) for categorical variables and Wilcoxon-Mann- Whitney test for continuous variables

### Respiratory support in the first 24 h and in-hospital treatment

The type of support used to treat respiratory failure in the first 24 h and in-hospital treatment are shown in Table 3. In order to correct hypoxemia, 34.8% (n = 40) of women required low flow oxygen nasal cannula, whereas in men this treatment was sufficient in a lower percentage (i.e. 23.7% [n = 71], *p* = 0.022). Indeed, males necessitated CPAP more frequently than females at presentation (25.7% vs 13.0%; *p* = 0.006). Considering in-hospital treatments, no gender differences were observed, with the exception of IL-6 pathway inhibitors that were used only in 17 males.

### Clinical outcomes and gender as predictor of mortality

**Fig. 1:**
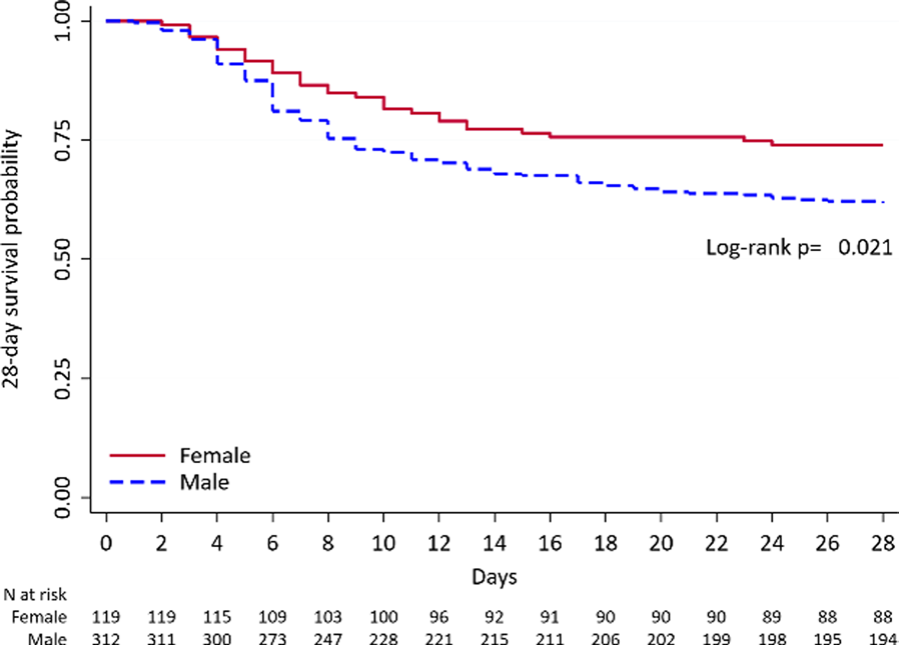
Kaplan–Meier 28-day mortality since hospitalization by gender in the overall population

Overall 28-day mortality occurred in 34.8% of the patients (150/431), of whom 26.1% (31/119) were women and 38.1% (119/312) were men (*p* = 0.018, Additional file 1: Table S2). Kaplan–Meier survival curve at 28-day (Fig. 1) found a lower mortality for females (*p* = 0.021). The secondary outcome (i.e. development of severe disease) were reported in the 59.9% of the patients (258/431), and it occurred more frequently in male patients (63.1% (197/431) *vs* 51.3% (61/11); *p* = 0.024, Additional file 1: Table S2). When outcomes were stratified by age categories, no gender differences were noted (*p* = 0.091 and *p* = 0.052 for primary and secondary outcome, respectively, Additional file 1: Table S2).

**Fig. 2:**
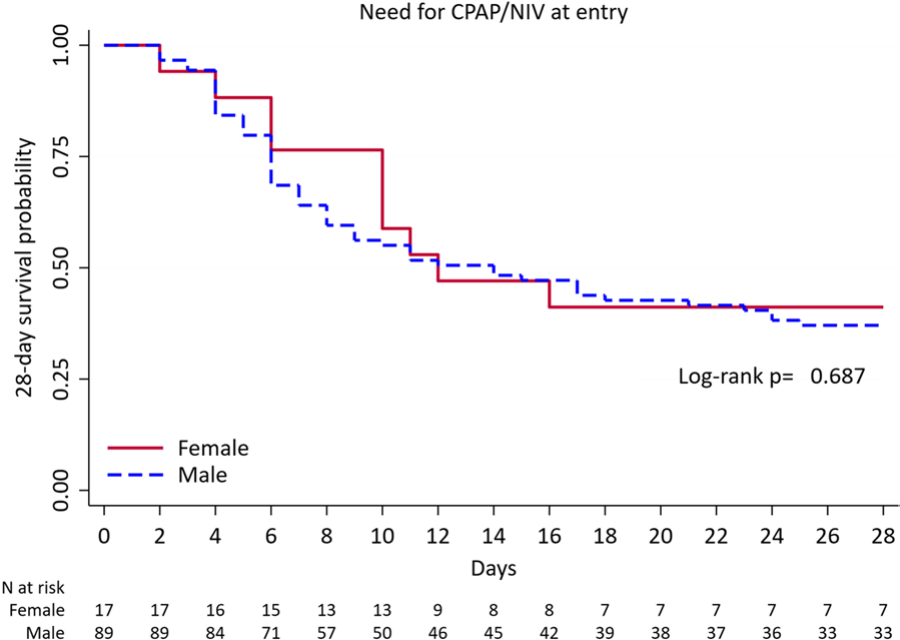
Kaplan–Meier 28-day mortality since hospitalization by gender in patients who needed CPAP/NIV in the first 24 h. *CPAP* Continuous positive airway pressure, *NIV* non-invasive ventilation

Multivariable analysis aimed at evaluating independent predictors of mortality is shown in Table 5. When baseline demographic, clinical characteristics and pre-hospital epidemiologic and clinical features emerged by univariate analysis were included in the multivariable analysis, male sex, together with older age, immunosuppression and dyspnea resulted independent predictors of death. However, once the parameters related to the severity of disease at presentation (i.e. need of CPAP or NIV in the first 24 h, and PaO_2_/FiO_2_ < 200 mmHg at admission) were included in the model, gender did not result an independent predictor of death (*p* = 0.898, Table 5). Accordingly, Kaplan–Meier survival analysis at 28-day in patients who needed CPAP or NIV in the first 24 h did not find a significant difference between men and women (Fig. 2, *p* = 0.687).

**Table 5:** Univariate and multivariable predictors of 28-day mortality since hospitalization

	Univariate model (all candidate predictors)		Multivariable model (selected predictors*)	
	OR (95% CI)	*p*	OR (95% CI)	*p*
Male sex	1.75 (1.10–2.80)	0.019	1.05 (0.47–2.37)	0.898
Age—y				
≤ 59	1.00 (Ref.)	–	1.00 (Ref.)	–
60–69	3.19 (1.40–7.29)	0.006	2.84 (0.91–8.86)	0.072
70–77	8.99 (4.14–19.54)	< 0.001	13.36 (4.39–40.60)	< 0.001
≥ 78	19.59 (8.89–43.15)	< 0.001	31.73 (9.29–108.44)	< 0.001
Former smoker	2.17 (1.31–3.58)	0.003	–	–
Hypertension	3.09 (2.00–4.78)	< 0.001	–	–
Diabetes				
No diabetes	1.00 (Ref.)	–	–	–
Diabetes, not insulin dependent	1.76 (0.98–3.13)	0.057	–	–
Diabetes, insulin dependent	3.51 (1.52–8.08)	0.003	–	–
Chronic Kidney Failure	2.56 (1.22–5.36)	0.012	–	–
COPD	1.98 (1.04–3.79)	0.039	–	–
Active hematologic malignancy	5.41 (1.41–20.72)	0.014	–	–
Previous Myocardial Infarction	3.23 (1.79–5.83)	< 0.001	–	–
Vasculopathy	3.18 (1.78–5.68)	< 0.001	–	–
ACE–inhibitors	2.09 (1.25–3.49)	0.005	–	–
OAT/DOACs	1.96 (1.08–3.54)	0.026	–	–
Antiplatelets	3.10 (1.98–4.87)	< 0.001	–	–
Immunosuppression	2.20 (1.01–4.82)	0.048	–	–
Flu vaccine	2.53 (1.51–4.25)	< 0.001	–	–
Symptoms onset—ER, per 1-day increase	0.92 (0.87–0.97)	0.001	–	–
Cough	0.61 (0.40–0.91)	0.016	–	–
Dyspnea before admission	1.66 (1.09–2.52)	0.018	–	–
Systemic symptoms	0.61 (0.39–0.96)	0.032	–	–
CPAP/NIV in the first 24 h	4.73 (2.97–7.53)	< 0.001	7.07 (3.20–15.61)	< 0.001
PaO_2_/FiO_2_ ratio < 200	3.73 (2.26–6.14)	< 0.001	3.05 (1.49–6.24)	0.002
Blood urea, per 1-unit increase	1.02 (1.01–1.02)	< 0.001	1.01 (1.00–1.02)	0.041

## Discussion

The main results from this study, aimed at evaluating the role of gender in Covid-19 hospitalized patients, can be summarized as follows. First, women are less prevalent than men in our setting, representing about a third of hospitalized male population. Second, both 28-day mortality and severe disease occur less frequently in women. Third, different mortality in sex categories cannot be ascribed to age per se. Fourth, once severe disease has occurred, the risk of dying from Covid-19 is not affected by gender.

The importance of the evaluation of sex- and gender-specific effects of Covid-19 has been recently emphasized, with the aim to develop approaches able to address the acute and long-term effect of the disease [19]. Availability and access to health care facilities, especially in low income countries, could be different for women and men [20]. As largely expected, in our cohort, we do not find any significant difference between gender categories in terms of pre hospital antibiotic treatment, home or professional exposure to confirmed Covid-19 cases and interval between symptoms onset and hospital presentation, suggesting an equitable access to healthcare. Our study population mainly consists of male individuals (72.4% vs 27.6%), with a male/female ratio of 2.6:1. Available Covid-19 literature shows variable sex prevalence depending on clinical setting. Epidemiological reports based on notification of infectious disease describe similar prevalence between sex categories [12, 21, 22]. On the other hand, when considering hospitalized Covid-19 population, a ratio of about 1.5:1 is found [9, 10, 23]. Moreover, male prevalence increases in ICU setting, ranging from 1.5 to 2.0:1 up to 4:1 in a recent Italian study [24–26]. Taken together, our study and other epidemiological data confirm a more severe disease in males. Furthermore, as a clue of this result, we found an exclusive use of IL-6 inhibitors only in few males with particular compromised clinical condition and relentless deterioration in gas exchanges in spite of optimized conventional therapy (Table 3). Considering mortality, women are significantly more likely than men to survive the infection, in accordance with recent literature on Covid-19 [9]. Of note, in order to standardize and valorise the analysis, we described mortality at 28-day since hospitalization, which is a shared and reasonable interval of time in acute settings.

In the case of Covid-19, an enzymatic system involved in this different sex predisposition could be represented by angiotensin converting enzyme 2 (ACE2), which allows penetration of SARS-CoV-2 into cells and is down-regulated by the virus [13]. ACE2 is counter regulatory to the activity of angiotensin II, leading to angiotensin-(1–7) formation, which exerts vasodilatory, anti-inflammatory, antifibrotic, and antigrowth effects. Animal model observations demonstrated a hormonal susceptibility of ACE2. In mice it has been shown that 17ß-estradiol increases the expression and activity of ACE2 while ovariectomy results in a decreased activity. Conversely hypertensive male mice have a higher myocardial ACE2 expression than females and its levels decreases after orchiectomy [27, 28]. Moreover, sex hormones can affect the immune and inflammatory modulation during infection, with estrogens promoting both innate and adaptive immunity and testosterone having a suppressive effect on immune function [29]. Actually, in our cohort, biochemical profile at presentation (i.e. platelets counts, coagulation, liver and renal function, CRP and PCT) suggests a tendency to a lower inflammatory status and organs impairment in females (Table 4). Finally, preliminary data have advocated a crucial role of endothelium in Covid-19. A role of estrogen (i.e. 17β-estradiol or E2) on vascular function and the endothelium have been suggested [30]. The mechanisms proposed include the generation of NO and prostacyclin, promotion of endothelial repair and regeneration, anti-inflammatory and antioxidant effects [31]. Our female population confirmed to be less fragile in this field, having few lifestyle risk factors (i.e. smoking history), and a lower rate of vasculopathy and myocardial infarction (Table 1). Thus, the lower severity of Covid-19 in women can be due to the influence of gender-related factors at least on: (1) the mechanism of cell entry of the virus; (2) the immune and inflammatory modulation during infection; (3) the endothelium and vascular function. Moreover, gastrointestinal symptoms at presentation, which were inconsistently correlated to outcome in previous reports, are more common in females (Table 2) [32–35]. This result could reflect the higher expression of ACE2 in colon transverse in females. As a matter of fact, a recent systematic survey showed that ACE2 presents remarkable differences in male–female expression levels possibly due to differences in escape from X inactivation [36].

Interestingly, in our multivariable analysis, sex does not result an independent predictor of death. Similar results were found during SARS in 2003, where a significantly higher mortality rate in males have been described, even if sex was not an independent predictor of mortality [37]. Specifically, when we add the severity of respiratory failure at presentation in the multivariable model (i.e. PaO_2_/FiO_2_ ratio < 200 mmHg at presentation and CPAP/NIV need in the first 24 h), it prevails on sex influence. The multivariable model is confirmed by survival analysis which demonstrates that male and female patients who required CPAP or NIV in the first 24 h have similar outcome (Fig. 2).

Our study has several limitations. First, it must be acknowledged that this is a retrospective study based on electronic medical records collected during a medical emergency, thus the accuracy of data may be reasonably questioned. We cannot exclude that missing data could have affected the significance of some variables. However, the effect of gender on mortality risk did not change (OR male vs. female = 1.39, 95% CI 0.76–2.45,* p* = 0.288) if we imputed missing values using multiple imputation by chained equation (MICE) with 20 imputation sets. Of note, this is a large series of cases coming from the forefront of the outbreak, addressing gender differences and providing a substantial follow-up length on hard endpoints. Secondly, our cohort consists of a large proportion of male patients, and this could have brought to an imbalance between represented sex categories. Moreover, research on sex and gender exceeds stratification of patients by these variables, needing the evaluation of biological (e.g. hormonal state, immune function and comorbidities), and gender-related factors (e.g. lifestyle and socioeconomic status) [19]. Prospective studies are required to better characterize patients by the evaluation of more specific sex- and gender-related parameters.

## Conclusions

Women admitted for Covid-19 have significantly few cardiovascular comorbidities and lifestyle risk factors, and more gastrointestinal symptoms prior to admission. Moreover, female patients present less severe disease and are more likely to survive the infection. However, once severe disease occurs, the risk of dying is similar regardless of gender.

## Supplementary Information

**Additional file 1: Supplementary Definitions. Table S1.** Intervals between symptoms and clinical relevant episodes in all patients and by gender. **Table S2**. 28-day outcomes according to gender and age.

**Additional file 2.** Covid-19 Study Group.

## Data Availability

The datasets generated and/or analysed during the current study are available in Papa Giovanni XXIII Hospital digital repository. The datasets generated and/or analysed during the current study are not publicly available due individual privacy policy but are available from the corresponding author on reasonable request.

## Abbreviations

ABG: Arterial blood gas analysis
ACE2: Angiotensin converting enzyme 2
ALT: Alanine aminotransferase
AST: Aspartate transaminase
CCI: Charlson Comorbidity Index
CI: Confidence intervals
Covid-19: Coronavirus disease 2019
CPAP: Continuous positive airway pressure
CRP: C Reactive protein
E2: Estradiol
ER: Emergency room
ETI: Endotracheal intubation
FiO_2_: Fractional inspired oxygen
HCO_3_^−^: Bicarbonate
HIV: Human immunodeficiency virus
ICU: Intensive Care Unit
IL-6: Interleukin-6
IQR: Interquartile range
MERS: Middle east respiratory syndrome
MICE: Multiple imputation by chained equation
NIV: Non-invasive ventilation
NO: Nitric oxide
OR: Odds ratio
PaCO_2_: Partial pressure of carbon dioxide
PaO_2_: Arterial oxygen partial pressure
PCT: Procalcitonin
RNA: Ribonucleic acid
RT-PCR: Real time polymerase chain reaction
SARS: Severe acute respiratory syndrome
SARS-CoV-2: Severe acute respiratory syndrome coronavirus 2
SD: Standard deviation
WHO: World Health Organization
