# Supplementary material for: Covid-19 and gender: lower rate but same mortality of severe disease in women—an observational study
Source: BMC Pulm Med. 2021 Mar 20;21:96. doi: 10.1186/s12890-021-01455-0 (PMC7980742; doi:10.1186/s12890-021-01455-0)
Supplement: Supplementary file 1 — Additional file 1: Supplementary Definitions. Table S1. Intervals between symptoms and clinical relevant episodes in all patients and by gender. Table S2. 28-day outcomes according to gender and age. [file 12890_2021_1455_MOESM1_ESM.docx]

**Covid-19 and gender: lower rate but same mortality of severe disease in women**

An observational study

Federico Raimondi^1,2^, Luca Novelli^1^, Arianna Ghirardi^3^, Filippo Maria Russo^2,4^, Dario Pellegrini^5^, Roberta Biza^1,2^, Roberta Trapasso^1,2^, Lisa Giuliani^1,2^, Marisa Anelli^1,2^, Mariangela Amoroso^1,2^, Chiara Allegri^1,2^, Gianluca Imeri^1^, Claudia Sanfilippo^1^, Sofia Comandini^1^, England Hila^2,4^, Leonardo Manesso^2,4^, Lucia Gandini^2,4^, Pietro Mandelli^2,4^, Martina Monti^2,4^, Mauro Gori^5^, Michele Senni^5^, Ferdinando Luca Lorini^4^, Marco Rizzi^6^, Tiziano Barbui^3^, Laura Paris^7^, Alessandro Rambaldi^2,7^, Roberto Cosentini^8^, Giulio Guagliumi^5^, Simonetta^9^ Cesa, Michele Colledan^10^, Maria Sessa^11^, Arianna Masciulli^3^, Antonello Gavazzi^3^, Sabrina Buoro^12^, Giuseppe Remuzzi^13^, Piero Ruggenenti^14^, Annapaola Callegaro^15^, Andrea Gianatti^16^, Claudio Farina^15^, Antonio Bellasi^17^, Sandro Sironi^18,19^, Stefano Fagiuoli^20^, and Fabiano Di Marco^1,2^*, on behalf of HPG23 Covid-19 Study Group

***Corresponding Author:** Prof. Fabiano Di Marco, M.D. Ph.D., Pulmonary Medicine Unit, Medicine Department, ASST Papa Giovanni XXIII, Piazza OMS, 1 - 24127 Bergamo, Italy. Professor of Pulmonary Medicine Università degli Studi di Milano. Tel. +39 035.2673456 - Fax +39 035.2674889 - Email: [fabiano.dimarco@unimi.it](mailto:fabiano.dimarco@unimi.it)

**APPENDIX MATERIAL**

**Supplementary Definitions:**

- Fever was defined as an axillary temperature of 37.5°C, or higher
- Peripheral vascular disease was defined as intermittent claudication or past bypass for chronic arterial insufficiency, history of gangrene or acute arterial insufficiency, or untreated thoracic or abdominal aneurysm (≥6 cm)
- Cerebrovascular disease was defined as medical history of stroke, transient ischemic attack, or head CT scan alterations consistent with previous ischemic injuries.
- Peptic ulcer disease was defined as any history of treatment for ulcer disease or history of ulcer bleeding.
- Immunosuppression: patients on chronic treatment with: steroids, antimetabolite drugs, calcineurin or mammalian receptor for rapamycin (mTOR) inhibitors (e.g. solid or hematologic transplanted patients), or patient with an established diagnosis of human immunodeficiencies virus (HIV).
- Other comorbidities (i.e. Hypertension, diabetes, chronic kidney disease, chronic obstructive pulmonary disease, active solid neoplasm, active hematological malignancy, cerebrovascular disease, previous myocardial infarction, chronic heart failure, rheumatic pathology) were included in our data when already reported in medical documentation in accordance with a previous specialist diagnosis.

**Appendix Table 1. Intervals between symptoms and clinical relevant episodes in all patients and by gender.**

|  | **N** | **All patients**  **(N=431)** | **Gender** | | |
| --- | --- | --- | --- | --- | --- |
|  |  |  | **Female (N=119)** | **Male (N=312)** | **p** |
| **Median days [IQR] between** |  |  |  |  |  |
| Symptoms onset – ER | 419 | 7.0 [5.0-10.0] | 8.0 [5.0-10.5] | 7.0 [5.0-10.0] | 0.97 |
| Symptoms onset – CPAP/NIV | 188 | 9.0 [6.5-11.0] | 10.0 [7.5-13.0] | 8.0 [6.0-11.0] | 0.080 |
| Symptoms onset – ETI | 76 | 10.0 [8.0-14.0] | 10.0 [9.0-16.0] | 10.0 [8.0-14.0] | 0.43 |
| ER - Hospitalization | 430 | 2.0 [1.0-2.0] | 2.0 [1.0-2.0] | 2.0 [1.0-2.0] | 0.27 |
| Hospitalization – CPAP/NIV | 192 | 1.0 [1.0-3.0] | 2.0 [1.0-4.0] | 1.0 [1.0-3.0] | 0.017 |
| Hospitalization – ETI | 77 | 4.0 [2.0-6.0] | 3.0 [1.0-6.0] | 4.0 [2.0-6.0] | 0.54 |
| CPAP/NIV – ETI | 70 | 3.0 [2.0-6.0] | 2.5 [2.0-5.5] | 3.5 [2.0-6.0] | 0.46 |

Continuous Positive Airway Pressure (CPAP), Non-invasive ventilation (NIV), Endotracheal Intubation (ETI), Median time between symptoms onset and the Emergency Room (ER) admission, between symptoms onset and CPAP/NIV initiation, between symptoms onset and ETI, between the ER admission and hospitalization in a ward, between hospitalization after the ER admission and CPAP/NIV initiation, between hospitalization after the ER admission and ETI, between CPAP/NIV initiation and ETI, IQR [Interquartile Range], p-values obtained by Wilcoxon-Mann-Whitney test for continuous variables.

**Appendix Table 2. 28-day outcomes according to gender and age.**

|  | **N** | **All patients**  **(N=431)** | **Gender** | |  |
| --- | --- | --- | --- | --- | --- |
|  |  |  | **Female** | **Male** | **p** |
|  |  |  | **(N=119)** | **(N=312)** |  |
| **Death** | 431 | 150 (34.8) | 31 (26.1) | 119 (38.1) | 0.018 |
| **Death by age** |  |  |  |  |  |
| ≤ 59 – *n (%)* |  | 9 (2.1) | 2 (1.7) | 7 (2.2) | 0.091 |
| 60-69 – *n (%)* |  | 23 (5.3) | 2 (1.7) | 21 (6.7) |  |
| 70-77 – *n (%)* |  | 51 (11.8) | 13 (10.9) | 38 (12.2) |  |
| ≥ 78 – *n (%)* |  | 67 (15.5) | 14 (11.8) | 53 (17.0) |  |
| **Severe disease*** | 431 | 258 (59.9) | 61 (51.3) | 197 (63.1) | 0.024 |
| **Severe disease* by age** |  |  |  |  |  |
| ≤ 59 – *n (%)* |  | 66 (15.3) | 17 (14.3) | 49 (15.7) | 0.052 |
| 60-69 – *n (%)* |  | 55 (12.8) | 8 (6.7) | 47 (15.1) |  |
| 70-77 – *n (%)* |  | 68 (15.8) | 21 (17.6) | 47 (15.1) |  |
| ≥ 78 – *n (%)* |  | 69 (16.0) | 15 (12.6) | 54 (17.3) |  |

Data expressed as column percentages, **Severe disease as described in Materials and Methods of main text*, p-values obtained by Chi-square test (or Fisher's exact test when appropriate).
