# Supplementary material for: Covid-19 and gender: lower rate but same mortality of severe disease in women—an observational study
Source: BMC Pulm Med. 2021 Mar 20;21:96. doi: 10.1186/s12890-021-01455-0 (PMC7980742; doi:10.1186/s12890-021-01455-0)
Supplement: Supplementary file 2 — Additional file 2. Covid-19 Study Group. [file 12890_2021_1455_MOESM2_ESM.docx]

**Members of the HPG23 Covid-19 study group (Papa Giovanni XXIII Hospital, Bergamo, Italy):**

Alimonti Dario, MD Neurology Unit

Amaduzzi Annalisa, MD Surgery Unit, Department of Organ Failure and Transplantation

Binda Francesca, MD Infectious Diseases Unit

Bombana Enrico, MD Infectious Diseases Unit

Bombardieri Giulia, MD Medical Healthcare Coordination Unit

Cacciabue Eleonora Marina, MD Head of Medical Healthcare Coordination Unit

Caldara Cristina Nurse in charge territorial service

Camera Giorgia, MD Neurology unit

Capelli Cinzia Bed manage, Healthcare Coordination Unit

Cappelletti Laura, MD Nephrology Unit

Carrara Camillo, MD Nephrology Unit

Colombi Renata, MD Emergency Medicine Unit

Cortinovis Fiorenzo, MD Nutrition and Dietology Unit

Daminelli Marinella, MD Nurse in charge, Pharmacy department

De Giorgio Massimo, MD Gastroenterology Hepatology and Transplantation Unit

De Vecchi Massimiliano, MD Emergency Medicine Unit

Del Prete Luca, MD Surgery Unit, Department of Organ Failure and Transplantation

Di Giorgio Angelo, MD Paediatric Hepatology Gastroenterology and Transplantation Unit

Duca Andrea, MD Emergency Medicine Unit

Ferrari Maddalena, MD Nurse in charge, operating room

Franca Averara, MD Infection prevention nurse

Gaffuri Giovanna, MD Gastroenterology Hepatology and Transplantation Unit

Ghilardi Patrizia, MD Nurse in charge maternal and pediatric department

Marchetti Marina, MD Immunohematology and Transfusion Unit

Micò Caterina, MD Department of Oncology and Hematology

Moraschini Fiorenzo, MD Nurse intensive care Unit

Pagani Gabriele, MD Medical Healthcare Coordination Unit

Piccichè Antonio , MD Medical Healthcare Coordination Unit

Portalupi Valentina, MD Nephrology Unit

Ripamonti Diego, MD Infectious Diseases Unit

Rodeschini Eleonora, MD Nurse in charge Ospedale San Giovanni Bianco

Rota Lauretta, MD Nurse in charge department urgency and emergency

Scetti Silvia, MD Medical Healthcare Coordination Unit

Sonzogni Aurelio, MD General Pathology Unit

Spada Chiara Nurse in charge Medical and oncology department

Tebaldi Alessandra, MD Infectious Diseases Unit

Tomasoni Laura, MD Nurse in charge Cardiovascular and surgery department

Tornese Stefania, MD Surgery Unit, Department of Organ Failure and Transplantation

Trevisan Roberto, MD Endocrinology and Diabetology Unit

Valoti Oliviero Francesco , MD Intensive care Unit 4

Zanotti Anna Nurse in charge logistics and patient transport
